# TreeGrid: A Spatial Planning Tool Integrating Tree Species Traits for Biodiversity Enhancement in Urban Landscapes

**DOI:** 10.3390/ani15131844

**Published:** 2025-06-22

**Authors:** Shrey Rakholia, Reuven Yosef, Neelesh Yadav, Laura Karimloo, Michaela Pleitner, Ritvik Kothari

**Affiliations:** 1 Bioinformatics Center, Forest Research Institute, Dehradun 248006, Uttarakhand, India; rakholias@gmail.com (S.R.); neelesh_yadav@icfre.org (N.Y.); ritvikkothari.cs2018@gmail.com (R.K.); 2 Eilat Campus, Ben Gurion University of the Negev, P.O. Box 272, Eilat 881020, Israel; 3 Faculty of Forest and Environment, Eberswalde University for Sustainable Development, 16225 Eberswalde, Germany; laura.karimloo@hnee.de; 4 Centre for Microbiology and Environmental Systems Science, University of Vienna, 1030 Vienna, Austria; michaela.pleitner@gmail.com

**Keywords:** biodiversity, ecosystem services, urban wildlife, urban heat island, urban forests

## Abstract

Urbanization, habitat fragmentation, and intensifying urban heat island (UHI) effects accelerate biodiversity loss and diminish ecological resilience in cities, particularly in climate-vulnerable regions. To address these challenges, we developed TreeGrid, a functionality-based spatial tree planning tool designed specifically for urban settings in the Northern Plains of India. The tool integrates species trait datasets, ecological scoring metrics, and spatial simulations to optimize tree placement for enhanced ecosystem service delivery, biodiversity support, and urban cooling. Developed within an R Shiny framework, TreeGrid dynamically computes biodiversity indices, faunal diversity potential, canopy shading, carbon sequestration, and habitat connectivity while simulating localized reductions in land surface temperature (LST). Additionally, we trained a deep neural network (DNN) model using tool-generated data to predict bird habitat suitability across diverse urban contexts. The tool’s spatial optimization capabilities are also applicable to post-fire restoration planning in wildland–urban interfaces by guiding the selection of appropriate endemic species for revegetation. This integrated framework supports the development of scalable applications in other climate-impacted regions, highlighting the utility of participatory planning, predictive modeling, and ecosystem service assessments in designing biodiversity-inclusive and thermally resilient urban landscapes.

## Simple Summary

Urbanization is causing biodiversity loss and harming city ecosystems. To address this issue at decision-making levels, we developed a planning tool for tree plantation in cities of the Northern Plains in India. This tool integrates tree data, spatial maps, and simulations representing a practical spatial decision support system for urban planners, city officials, environmentalists, and policymakers. It quantifies biodiversity potential, shade provision, carbon sequestration aspects, and cooling effects of trees in an urban setting. It employs artificial intelligence to predict areas with high potential for avian habitats. The results show that strategic tree planting can support wildlife, reduce heat, and enhance the connection between green areas.

## 1. Introduction

Planting trees in urban areas is a crucial strategy for attracting and sustaining wildlife, thereby enhancing urban biodiversity and ecological resilience. Trees serve as critical habitat elements, offering food resources, nesting sites, shelter, and movement corridors for various bird, insect, and mammal species [1,2]. Selecting native or ecologically functional tree species can significantly enhance urban wildlife richness and abundance by offering key traits that are favorable to invertebrates, such as nectar production, fruit availability, or bark textures [3]. Additionally, complex tree canopy structures promote vertical habitat layering, a factor positively associated with greater faunal diversity [4]. Therefore, integrating tree planting into urban design with a focus on species traits that enhance habitat quality is critical for fostering self-sustaining urban ecosystems and reducing the ecological degradation caused by habitat fragmentation.

The concept of wildlife-inclusive urban design involves integrating biodiversity aspects into urban design at an early stage, which can lead to significantly beneficial biodiversity outcomes [5,6]. Human-dominated urban landscapes often require spatial planning tools to manage human–wildlife conflict arising from foraging attractions in urban interfaces [7]. Public participatory tools, such as PPGIS, have been proposed to address planning deficits. Tools like PPGIS assist in identifying desirable park features, providing redesign suggestions, identifying conflict areas, and enabling planning to enhance the values of these features at the local level [8]. A robust conceptual participatory framework was developed to align species traits with urban spatial parameters. This framework emphasizes ecological compatibility alongside socio-cultural dimensions of human–wildlife interactions, such as observability (e.g., bird songs, seasonal appearances), ecosystem services (e.g., pollination by bees, pest control by bats), and cultural symbolism or potential conflicts [9].

The importance of spatial context in strategic tree planting is often underestimated. However, spatial considerations are crucial for optimizing available space and have a direct impact on the delivery of local ecosystem services [10]. Local ecosystem services, including street trees, urban forests, ponds, and farms, must be internalized in land-use planning, as they significantly affect living quality [11,12]. Tools like the CityTree model highlight how species-specific and size-dependent traits influence ecosystem services such as shading, transpiration, and carbon storage, which are relevant under all climate change scenarios [13].

Functionality-focused tools are essential in land-use planning for spatially quantifying the benefits of restored ecosystem services in urban areas. These services, including cooling, biodiversity, and social well-being, are often excluded from conventional cost–benefit analyses [14,15]. Recent tools such as the “Right Place, Right Tree” initiative in Boston have improved tree plantation decision-making by mitigating the impacts of urban heat islands [16]. The “Which Plant Where” tool represents another functionality-based approach. It integrates tree species trait data derived from the literature, stakeholder input, and experimental validation to assess co-benefits, including shading, carbon sequestration, faunal support, and canopy coverage, thereby optimizing ecological performance in urban areas [17].

The Convention on Biological Diversity (CBD) had already introduced the City Biodiversity Index (CBI) as a comprehensive assessment of urban biodiversity, which, in order to address the issue of habitat fragmentation, emphasized connectivity measures and the associated metrics to influence city planners in improving the connectivity of natural habitats [18]. In addition, trees outside forests play a crucial role in maintaining ecological connectivity, and even slight canopy loss or fragmentation can drastically affect the dispersal of forest plants, pollen, birds, mammals, and insects [19].

Furthermore, species-specific traits such as shade are particularly important in human-modified landscapes, where canopy cover enhances mammalian diversity and richness [20]. More importantly, the participatory framework should inevitably include private urban forests or residential trees, which contribute to around one-third of the species not found in public inventories and exhibit distinct service-based traits that influence faunal biodiversity. As urban forests exhibit higher variations in the functional traits of tree species, this enhances biodiversity [21].

Nevertheless, urbanization is contributing to biodiversity loss and degrading urban ecosystems [22]. A recent study linked urban expansion to habitat loss for up to 40% of terrestrial vertebrate species by 2050. Urban heat islands (UHIs) exacerbate land surface temperature (LST), intensifying thermal stress in urban areas [23]. Subsequent research has identified LST as a key driver of climate-related biodiversity loss, underscoring the need for integrated land management to mitigate its impacts [24]. In arid regions, rising LST has been shown to correlate with increased thermal stress and reduced precipitation, disrupting entire ecosystems [23]. Strategies such as sustainable land management and reforestation can help stabilize local climates and protect biodiversity [25]. Therefore, developing tools to mitigate LST is essential, especially in climate-vulnerable regions. To support trade-offs among complex and diverse objectives, such as ecosystem services, species suitability, and trait compatibility, deep neural networks (DNNs) can be effectively applied. By maximizing multi-criteria performance through learning nonlinear relationships and optimizing tree placement for diverse spatial and ecological contexts, DNNs provide a robust and practical approach. Another recent study applied DNNs to optimize urban tree placement and increase urban tree connectivity, ensuring sufficient habitat patches for birds, insects, and other faunal groups. Hence, utilizing DNNs will provide better biodiversity prediction as it is a key indicator of ecosystem health. DNNs can effectively estimate the impact of environmental variables on ecosystem services and sustainability, enabling stronger planning at the ecosystem level [26,27,28].

Additionally, the “*Diversity4Restoration*” tool, which comprehensively encompasses species selection optimization, ecosystem services, planting diversity, climate resilience, and biodiversity restoration, can be considered for developing a similar tool for the Northern Indian Plains. This tool, when adapted to the unique ecological conditions of the Northern Plains, can significantly contribute to urban biodiversity planning and restoration efforts in the region. Building upon recent advances in urban biodiversity planning, ecological connectivity modeling, and climate adaptation strategies, this study aims to develop an integrated, functionality-focused urban tree planning tool tailored for the Northern Plains of India [29,30,31]. Specifically, the objectives are as follows:

- Integrating a biodiversity planning module into a spatial tree placement and cooling effect simulation tool will enable dynamic assessments of ecosystem service delivery, habitat suitability, and spatial ecological connectivity within fragmented urban landscapes.
- The tool’s data will be utilized to train deep neural networks (DNNs) to predict habitat connectivity and suitability for urban wildlife species. This will allow high-resolution mapping of biodiversity hotspots, support strategic greening interventions, and strengthen landscape-scale conservation planning efforts.

Ultimately, this study aims to contribute a scalable and adaptive approach to urban greening and biodiversity restoration. By emphasizing climate-vulnerable and ecologically fragmented urban regions, this tool has the potential to make a significant impact and inspire optimism for the future of urban biodiversity.

## 2. Materials and Methods

We developed the TreeGrid tool using a dataset comprising tree species native to and commonly found in the Northern Plains of India. This section outlines how we developed the tool to incorporate spatial visualization features and its subsequent applications in simulation and modeling using the generated data.

### 2.1. Tool Description

This spatial planning tool, viz. TreeGrid, developed within the R Shiny framework, is characterized by its distinct feature, the careful selection of tree species traits to develop ecological indices and compute relevant values. These traits, including tree height, canopy width, leaf area index, faunal groups, and ecosystem services score, form the basis for several spatially linked indices. A distinguishing feature of the tool is its ability to place trees on a spatial map and generate circles or canopy outlines based on each species’ estimated canopy width. Moreover, an automatic tree placement feature has also been added to the “Autogrid layout” tab. The Autogrid layout offers an automated method for placing trees within a user-drawn polygon. Once the user specifies the species and their intended counts, the tool generates a grid of potential planting points and strategically fills them, ensuring no spatial overlaps. Additionally, we derived mean LST data of the last five years (spatial resolution after resampling was 30 m) for the study area from Landsat 8–9 Thermal Infrared bands, processed via the Google Earth Engine cloud platform.

### 2.2. Ecological Score Computations

#### 2.2.1. Tree Plantings Biodiversity Score

For species richness, the widely adopted 10:20:30 rule by Santamour was explicitly applied to the planting scheme. According to this guideline, no more than 10% of any single species, 20% of any genus, and 30% of any family should be planted, thereby promoting biodiversity and resilience in urban forests [32,33]. Species evenness is assessed using Pielou’s Evenness Index (J), which quantifies how uniformly individual trees are distributed among the selected species. This index is calculated based on the relative abundance of each species in the planting layout, regardless of whether the placement is manual or automated. A perfectly even distribution (i.e., all species equally represented) yields a score close to 1, whereas dominance by a few species results in lower values.

#### 2.2.2. Faunal Biodiversity Score

Each tree species was assigned a faunal biodiversity score, ranging from 0 to 5, based on its documented support for five faunal groups: pollinators, birds, insects, lizards, and mammals. The scoring considered each species’ capacity to provide food, shelter, and habitat resources for the relevant faunal groups. This method and dataset for some species were adapted from the Which-Plant–Where methods of biodiversity score computation. Additionally, local validation was utilized from the Forest Research Institute’s preexisting datasets, such as http://www.haryanaforestflora.in/ (accessed on 20 April 2025).

#### 2.2.3. Shade Index

Lateral canopy shading was estimated using the methodology developed by the “Which-Plant–Where” project as part of its co-benefits framework. This involves multiplying the maximum canopy width (CW) by the maximum tree height (TH), as shown in Equation (1) [17].

Shade Index = CW × TH(1)

#### 2.2.4. Carbon Value

Carbon sequestration potential was estimated by categorizing TH into low, medium, and high tiers based on the 33rd and 66th percentiles of the height distribution. This tertile-based approach provides a relative measure of carbon storage capacity, with taller trees assumed to contribute more significantly to long-term carbon accumulation [17].

#### 2.2.5. Ecosystem Services Score

We quantified the ecosystem services by counting the number of distinct services associated with each tree species as listed in the data tables. These services included air quality improvement, biodiversity support, bird feeding, carbon sequestration, erosion control, pollination, pest suppression, runoff reduction, and urban heat island (UHI) mitigation, among others. Based on the total count of these services per species, we categorized the trees into three categories: Low (1–3 services), Moderate (4–6 services), or High (>7 services) ecosystem service contributors.

#### 2.2.6. Connectivity Index

The connectivity index was computed by evaluating pairwise Haversine distances between all planted trees to identify those within a specified threshold (i.e., 50 m), indicating potential for ecological interaction or movement corridors. It is expressed as the ratio of connected tree pairs to the total possible pair combinations, reflecting the spatial cohesion of the planting layout. We performed this calculation using the *geosphere* package in R4.5.0. The Haversine method is commonly employed for point-based connectivity analysis and is widely used in geospatial navigation applications [34].

#### 2.2.7. Cooling Index

The cooling index (CI) estimates the temperature reduction (in °C) associated with each tree species based on its height, crown diameter, and leaf area index (LAI), which collectively influence shading and evapotranspiration. These variables are normalized and weighted (40% tree height, TH; 20% tree canopy width, CW; 40% LAI), then scaled to represent realistic urban cooling effects ranging from 2 °C to 8 °C (Equation (2)). This was developed based on a meta-analysis of research on key tree traits, specifically tree height, crown diameter, and LAI, which drive urban temperature reduction [35]. We subsequently used the calculated cooling index values to simulate spatial temperature reductions across land surface temperature (LST) raster grids.

CI = 2 + 6 × (0.4 × TH × 0.2 × CW + 0.4 × LAI)(2)

### 2.3. Spatial Cooling Effect Simulation

We simulated the spatial cooling effect using LST raster data in combination with the cooling index (CI) values assigned to each tree species. We employed the *Terra* R package 4.5.0 [36] to manipulate raster pixels and visualize localized LST reduction resulting from tree placements, estimating temperature reduction in degrees Celsius.

### 2.4. Predictive Habitat Modelling Based on Deep Neural Network

A DNN model was developed to predict bird habitat suitability using the *keras* library in R [37]. The training dataset comprised planted tree points with corresponding ecological attributes, including LST (°C), cooling index, shade index, height, and diameter. A binary label indicating the presence of birds was generated by checking whether “Bird” appeared in the biodiversity column and used as the response variable. All predictor variables were normalized, and the DNN architecture consisted of two hidden layers with ReLU (Rectified Linear Unit) activation followed by a sigmoid output layer for binary classification. The DNN was trained for 150 epochs using a batch size of 10, with an 80:20 training:validation data split. The trained model was used to make spatial predictions over the entire study area’s LST raster. The model’s performance across epochs was monitored using the loss and accuracy metrics, with both training and validation curves plotted to visualize learning dynamics and detect potential overfitting trends.

The trained model was used to make spatial predictions over the entire LST raster of the study area, where missing features were filled using the mean values from the training data. The predicted probabilities were rasterized to create a continuous surface representing the likelihood of bird hotspots across the landscape. A threshold of 0.5 was applied to classify high-probability zones as potential bird habitat hotspots for visualization. The *terra* and *sf* packages were used to convert between raster, point, and vector formats, and the leaflet was used for spatial visualization over satellite base maps.

## 3. Results

### 3.1. Tool Interface and Ecological Scores

**Figure 1:**
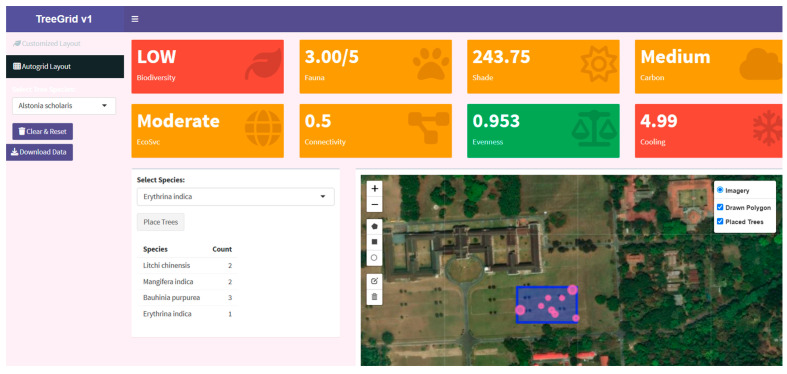
Interface of the TreeGrid Tool showing value boxes of biodiversity and ecological scores. Two layouts are shown: customized layout for individual tree placement and Autogrid layout for automatic placement of multiple selected trees.

The tool features an interactive interface that allows users to place tree buffers (represented as yellow circles) on a satellite base map using the Leaflet framework. For demonstration purposes, the map was centered on the Forest Research Institute in Dehradun. The dashboard displays key ecological metrics derived in real time based on the placement of selected tree species. The sidebar enables species selection, placement, data export, and visual feedback, while the top panel dynamically updates biodiversity and ecological metrics in response to the placement of trees (Table 1). If the user clicks inside the buffer of a tree, a warning appears, such as “conflict detected”. This feature is designed to prevent canopy overlap, thereby maintaining spatial accuracy and tool reliability. Users can download the added tree species list as training data for the DNN module in CSV format (Figure 1).

Additionally, the Autogrid layout feature inherently supports better species balance and spatial regularity and is particularly effective for large-scale or time-constrained planning. By design, Autogrid helps achieve high species evenness and efficient space utilization without requiring manual intervention for each placement, making it ideal for systematic biodiversity-oriented plantation schemes.

The computed metrics and their utilization are summarized in Table 1.

**Figure 2:**
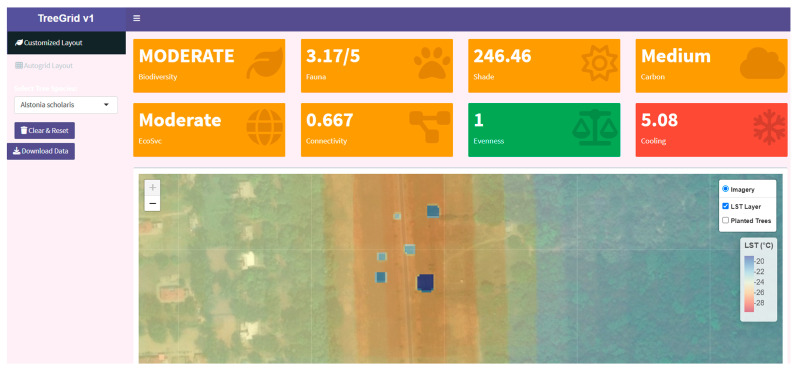
LST change simulation—map showing the cooling effect of the trees upon placement.

For temperature reduction simulation, the map visualizes planted trees as circular buffers based on their diameter (canopy size), overlaying a heat map of LST. When trees are placed, the change is simulated on the pixels under the tree buffers. The updated raster is rendered using *leaflet* and *Terra*, highlighting temperature gradients and the spatial cooling contribution of trees across the landscape. Users have complete control over the tool, being able to toggle base layers (Imagery, LST Layer, and Planted Trees) and export the session data for model training or further analysis. This level of control puts the user in charge of the tool’s functionalities, allowing them to adapt it to their specific needs (Figure 2).

**Figure 3:**
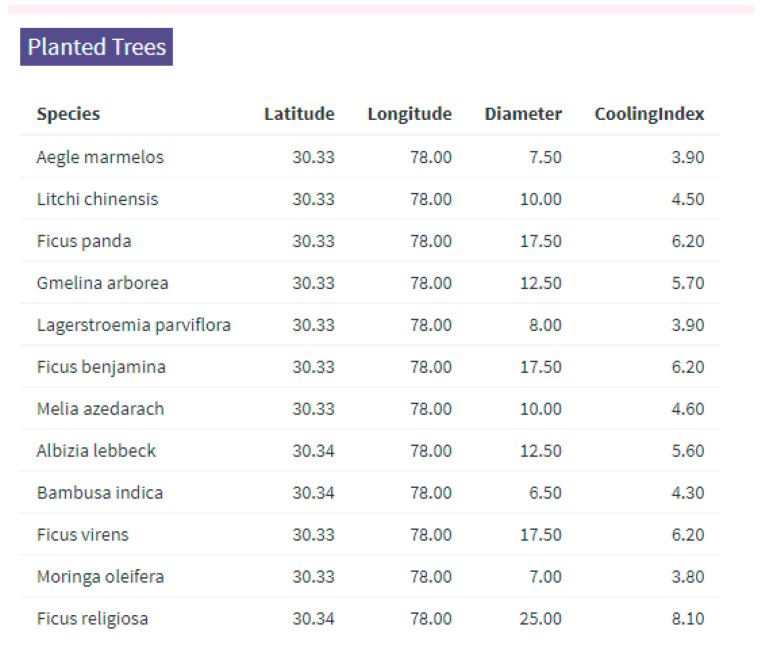
Summary list of tree species added for demonstration of figures. The latitude and longitude are in degree decimals and the diameter is in unit meters.

The lists of tree species are also instantly rendered from the Shiny application interface, along with their geospatial coordinates (latitude, longitude), estimated canopy diameter (size, in meters), and associated CI values. These values drive the spatial LST modification and the ecosystem service scoring within the tool (Figure 3).

The above visualization results display the working interface of the spatial tool, featuring easy-to-use features for planners.

### 3.2. Model Performance and Output

The performance of the DNN model, particularly its ability to learn from ecological inputs, is demonstrated below. The training loss (shown in the top panel, blue line) steadily decreases, indicating that the model is effectively learning from the input features derived from tree attributes and localized environmental data. Notably, the training accuracy (shown in the bottom panel, blue line) improves significantly after epoch 120, reaching nearly 95%. This suggests that the model has successfully captured complex patterns within the training set, instilling confidence in its predictive capabilities.

**Figure 4:**
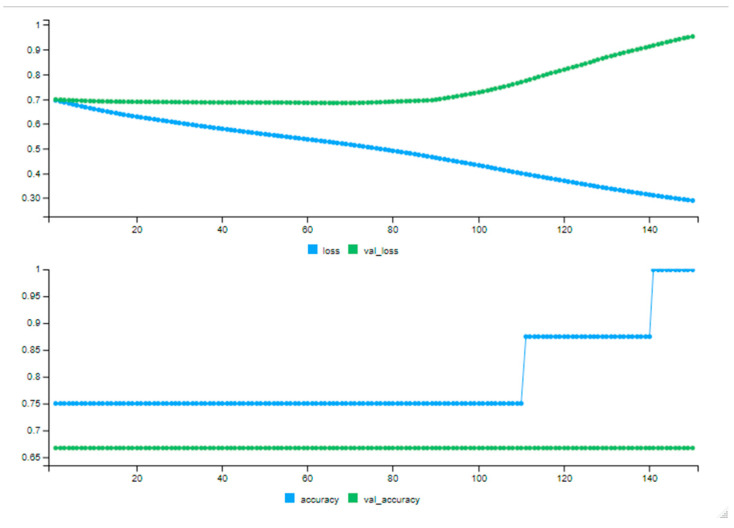
Training performance of the DNN model across 150 epochs for predicting bird habitat presence.

Although the validation loss (top panel, green line) rises modestly after approximately epoch 75, the validation accuracy (bottom panel, green line) remains consistent. This stability indicates that the model avoids erratic predictions on unseen data (Figure 4). These results underscore the model’s significant contribution to predicting spatial biodiversity, instilling optimism about its potential impact. The model’s ability to effectively learn from ecological variables sets the stage for fine-tuning and potential generalization with larger, more diverse datasets.

**Figure 5:**
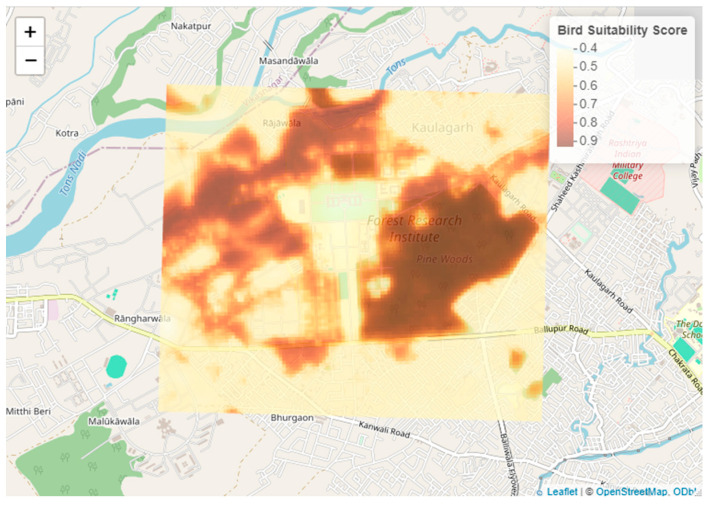
Predicted bird habitat map generated using a DNN model.

The heat map illustrates the distribution of bird suitability scores across the Forest Research Institute campus and its surrounding areas in Dehradun, India (Figure 5). The scores range from 0.4 to 0.9, with scores below 0.5 indicating low habitat suitability and scores above 0.8 indicating high habitat suitability for birds, as shown by the gradient color bar. Warmer colors, ranging from deep red to brown, highlight areas with higher predicted suitability. These areas often correspond to regions with denser vegetation, larger tree diameters, and favorable microclimates, which are influenced by previous tree placements and modifications in land surface temperature (LST).

**Figure 6:**
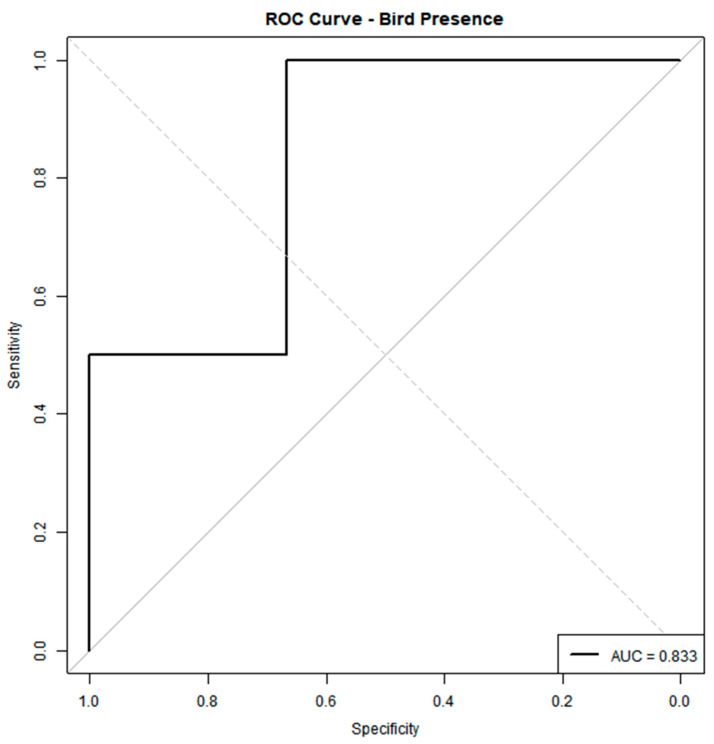
Area under curve/receiver’s operating characteristics.

The ROC curve for our DNN classifier (Figure 6) yields an AUC of 0.833, indicating good discrimination between points with and without bird presence. At most thresholds, the model achieves high specificity while maintaining a reasonable sensitivity, as evidenced by the steep rise of the curve toward the upper left corner. In particular, the classifier can accurately identify a large proportion of actual bird presence locations with relatively few false positives. An AUC above 0.8 suggests that our combination of LST, CI, shade index (SI), TH, and CS (diameter) captures relevant habitat signals. However, there remains room to improve sensitivity at lower false-positive rates.

## 4. Discussion

The TreeGrid facilitates animal movement within urban forest patches, a key aspect of biodiversity conservation. In tropical regions, where land scarcity prevents physical connectivity, urban forest fragmentation limits the survival of medium- and large-sized mammals. In such cases, ensuring functional connectivity becomes even more vital [35,38]. In tropical regions, where urban forest fragmentation limits the survival of medium- and large-sized mammals, especially when physical connectivity is no longer possible due to land scarcity, functional connectivity becomes even more critical [39]. The connectivity feature of our tool addresses this challenge by promoting functional habitat continuity through the spatial optimization of tree placement, thereby supporting wildlife movement across fragmented urban landscapes. By selecting the right tree species and providing key resources for fauna, such as nesting sites and bird food, suitable or surrogate habitats can be effectively created to compensate for lost natural habitats [40,41].

Other studies have demonstrated the importance of tree canopy size in supporting biodiversity and regulating urban microclimates, often visualized using circular buffer analyses. Based on these findings, future enhancements could involve strategic placement of shade trees using dynamic seasonal models, such as ENVI-met, to assess localized UHI mitigation [42]. Urban trees reduce daytime temperatures and enhance thermal comfort through evapotranspiration and shading effects [43]. Strategic tree placement mitigates localized UHI effects, reduces energy demands for cooling, and improves human health and well-being [44]. Our tool leverages real-world land surface temperature (LST) data collected from diverse urban settings as a baseline for simulating spatial cooling effects based on canopy characteristics. This aids urban planners in designing thermally resilient landscapes, emphasizing the critical role of tree spatial configuration in maximizing urban cooling benefits.

Furthermore, wildfires pose a significant threat to natural areas near cities, especially within wildland–urban interfaces (WUIs), where proximity to vegetation increases the risk of fire. Post-fire restoration strategies often involve planting native and endemic trees to aid in revegetation and restore ecosystem services critical for wildlife survival, such as providing shelter and food resources [45,46]. Our tool can assist in planning revegetation efforts in WUI areas, promoting the establishment of more resilient and biodiverse forests while reducing vulnerability to future fires. Specifically, the tool can be used to identify suitable species for planting, determine optimal spatial distribution, and assess the potential impact on biodiversity and ecosystem services. Additionally, planting strategies that account for species-specific traits and spatial distribution are crucial for maintaining ecosystem services and enhancing the structural complexity necessary for wildlife recolonization [47].

A key innovation of this study is the integration of deep neural networks (DNNs) to predict spatial habitat suitability based on tool-generated ecological data and user-defined parameters. By capturing complex nonlinear environmental relationships, deep learning models have shown significant promise in biodiversity modeling across extensive landscapes [48]. DNNs enable high-resolution predictions of habitat suitability and biodiversity hotspots, providing spatially actionable information crucial for urban biodiversity conservation planning. This approach aligns with emerging trends in conservation technology, where integrating AI with remote sensing and environmental datasets transforms species distribution modeling and habitat connectivity assessments [49].

The tool can also be integrated into citizen science initiatives to expand biodiversity data collection and promote public engagement. When combined with AI-driven tools, citizen science expands the spatial and temporal scope of data collection while raising awareness and fostering a stronger connection between urban residents and their local ecosystems [50]. This participatory model could enhance the tool’s impact by involving local communities in biodiversity mapping and urban greening initiatives, ultimately supporting more inclusive and effective conservation outcomes. By engaging urban residents in these initiatives, the tool not only enhances their understanding of local biodiversity but also fosters a sense of ownership and responsibility towards their urban environment.

Thus, by strategically combining ecological modeling, spatial planning, machine learning, and participatory approaches, this tool provides a comprehensive and scalable framework for enhancing urban biodiversity, mitigating urban heat stress, and promoting climate-resilient urban ecosystems. The spatial and computational approach provided by this tool, and its unique features like Autogrid, distinguish it from existing tools, which, despite having extensive datasets, have limited logic and model functionalities. Its scalability means that it can be applied to a variety of urban landscapes, from small urban forest patches to large metropolitan areas, making it a versatile and powerful tool for urban biodiversity planning and conservation efforts.

## 5. Conclusions

This study introduces an integrated spatial planning tool designed to tackle the pressing issues of urban biodiversity loss, habitat fragmentation, and microclimate regulation through strategic tree placement. By combining species trait databases, ecological service scoring, conflict-free placement strategies, land surface temperature (LST)-based cooling simulations, and deep neural network (DNN)-based habitat suitability predictions, the tool offers a novel, functionality-focused approach for enhancing biodiversity and urban resilience. The inclusion of connectivity metrics further enhances its effectiveness in mitigating the impacts of fragmentation in urban and peri-urban landscapes. The tool can also be integrated into citizen science initiatives to expand biodiversity data collection and promote public engagement. Leveraging deep learning to convert ecological data into spatial insights, the tool contributes to forward-looking, biodiversity-inclusive urban planning strategies in ecologically sensitive areas. However, it will be essential to address the practical implementation of tools at decision-making levels among stakeholders in the future.

## Figures and Tables

**Table 1:** Table showing results summary, i.e., computed metrics, respective range, and interpretation and utility.

Biodiversity and Ecology Metric	Range	Interpretation and Utility
Biodiversity Score (Trees)	Low/Moderate/High	Indicates tree species/genus/family diversity; higher score improves ecosystem resilience (species richness and evenness)
Faunal Diversity (Faunal Groups)	0–5	Reflects potential to support multiple faunal groups; higher values support richer biodiversity
Shade Index	0–875 (species-dependent)	Estimates shading potential based on tree height and canopy width; useful for thermal comfort planning
Carbon Value	Low/Medium/High	Indicator for biomass and long-term carbon sequestration potential
Ecosystem Services	Low (1–3), Moderate (4–6), High (7+)	Captures multifunctional value of an ecosystem; more services mean more co-benefits
Connectivity Index	0–1	Evaluates spatial cohesion of planting; helps maintain faunal movement corridors
Cooling Index	2–8 °C	Estimates temperature reduction; useful for UHI mitigation
LST Reduction Simulation	Contextual (1–5 °C based on placement and density)	Visualization of spatially simulated LST changes; aids urban microclimate design

## Data Availability

https://rakholias.shinyapps.io/TreeGridv1/, accessed on 13 June 2025.

## Abbreviations

AUC	Area under curve
CBD	Convention for Biodiversity
CBI	City Biodiversity Index
CI	Cooling index
CW	Maximum canopy width
DNN	Deep neural network
LAI	Leaf area index
LST	Land surface temperature
PPGIS	Participatory Planning Geographical Information Systems
ROC	Receiver operating characteristic (curve)
ReLU	Rectified Linear Unit
SI	Shade index
TH	Maximum tree height
UHI	Urban heat island
WUI	Wildland–urban interface
